# Host defence peptide LEAP2 contributes to antimicrobial activity in a mustache toad (*Leptobrachium liui*)

**DOI:** 10.1186/s12917-023-03606-3

**Published:** 2023-02-11

**Authors:** Jie Chen, Chi-Ying Zhang, Jing-Yi Chen, Rachel Wan Xin Seah, Le Zhang, Li Ma, Guo-Hua Ding

**Affiliations:** 1 Laboratory of Amphibian Diversity Investigation, College of Ecology, Lishui University, Lishui, 323000 China; 2 Department of Biological Science, National University of Singapore, Singapore, 117558 Singapore; 3 School of Medicine, Lishui University, Lishui, 323000 China

**Keywords:** Amphibian, Aantibacterial activity, Aantibacterial mechanism, Ggene expression, Lliver-expressed antimicrobial peptide 2

## Abstract

**Background:**

The liver-expressed antimicrobial peptide 2 (LEAP2) is essential in host immunity against harmful pathogens and is only known to act as an extracellular modulator to regulate embryonic development in amphibians. However, there is a dearth of information on the antimicrobial function of amphibian LEAP2. Hence, a LEAP2 homologue from *Leptobrachium liui* was identified, characterized, and chemically synthesized, and its antibacterial activities and mechanisms were determined.

**Results:**

In this study, LEAP2 gene (*Ll-LEAP2*) cDNA was cloned and sequenced from the Chong’an Moustache Toad (*Leptobrachium liui*). The predicted amino acid sequence of Ll-LEAP2 comprises a signal peptide, a mature peptide, and a prodomain. From sequence analysis, it was revealed that Ll-LEAP2 belongs to the cluster of amphibian LEAP2 and displays high similarity to the Tropical Clawed Frog (*Xenopus tropicalis*)‘s LEAP2. Our study revealed that LEAP2 protein was found in different tissues, with the highest concentration in the kidney and liver of *L. liui*; and *Ll-LEAP2* mRNA transcripts were expressed in various tissues with the kidney having the highest mRNA expression level. As a result of *Aeromonas hydrophila* infection, *Ll-LEAP2* underwent a noticeable up-regulation in the skin while it was down-regulated in the intestines. The chemically synthesized Ll-LEAP2 mature peptide was selective in its antimicrobial activity against several in vitro bacteria including both gram-positive and negative bacteria. Additionally, Ll-LEAP2 can kill specific bacteria by disrupting bacterial membrane and hydrolyzing bacterial gDNA.

**Conclusions:**

This study is the first report on the antibacterial activity and mechanism of amphibian LEAP2. With more to uncover, the immunomodulatory functions and wound-healing activities of Ll-LEAP2 holds great potential for future research.

## Background

Antimicrobial peptides (AMPs), which are a class of small peptides, are highly abundant and diverse in the innate immune system of amphibians [1]. AMPs are characterized by short amphiphilic peptides which are generally cationic, and they play an essential role in protecting the organisms from pathogenic bacteria [2–5], parasites [6], and fungi [7–10]. Some strains of pathogenic bacteria have major negative implications on the survival of the species, and AMPs are likely key players in host resistance of these pathogens [2–10]. Additionally, AMPs are also involved in extensive immunomodulatory functions [11, 12] well as healing skin wounds [13, 14].

In 2003, the liver-expressed antimicrobial peptide 2 (LEAP2) was first discovered and it was also the second blood-derived antimicrobial peptide [15]. It is primarily expressed in the liver and its structure consists of a cysteine-rich peptide and it possesses four conserved cysteine residues [15–17]. LEAP2 has been shown to destroy a variety of pathogenic microorganisms, including *Bacillus megaterium*, *Bacillus subtilis*, *Neisseria cinerea*, and *Micrococcus luteus* [15, 17]. Besides mammals, LEAP2 has been identified in different species of birds [18], reptiles [19], and even fishes [20, 21].

To date, the amphibian *LEAP2* gene was only reported in the African Clawed Frog (*Xenopus laevis*) [22]. LEAP2 of X. laevis was found to act as an extracellular modulator to regulate embryonic development [22]. However, there is a dearth of information on the antimicrobial function of amphibian LEAP2. Hence, we identified, characterized, and chemically synthesized another amphibian LEAP2, named Ll-LEAP2 from the Chong’an Moustache Toad *Leptobrachium liui* (Pope, 1947) (Anura: Megophryidae), which is an endemic species distributed in mountainous stream habitats of eastern China, and determined its antibacterial activities and mechanisms.

## Results

### Molecular characterization

**Fig. 1:**
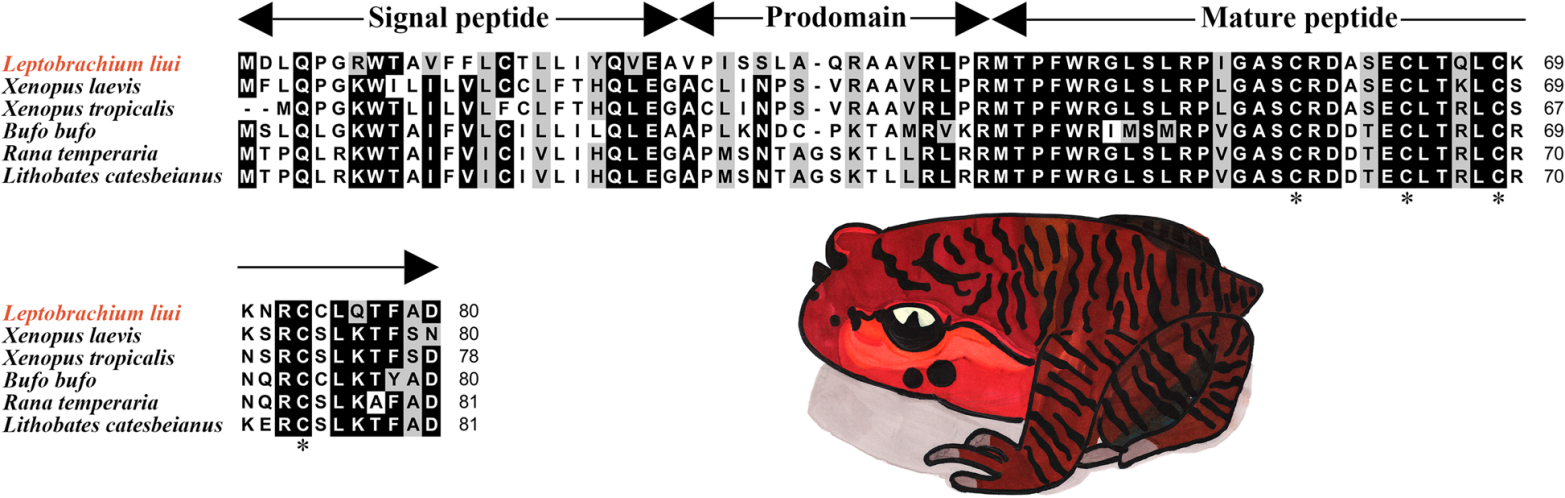
Multiple alignment of the amino acid sequences of Ll-LEAP2 and its homologs. The threshold for shading was 70%; similar residues are marked in grey, identical residues are marked in black, and alignment gaps are marked as “-”. The four conserved cysteine residues in the mature peptide are indicated by “*”. A cartoon picture of the experimental animal was painted by DING Zimu

**Fig. 2:**
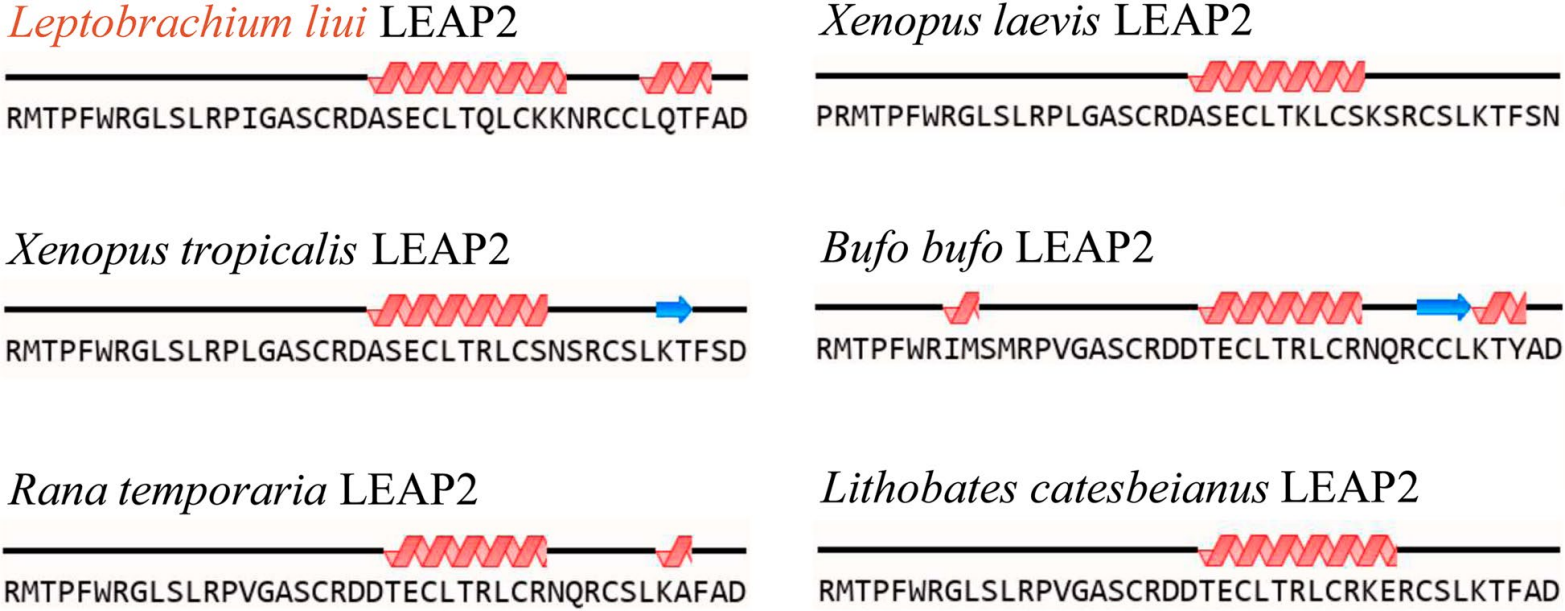
Amino acid sequences and predicted secondary structures of Ll-LEAP2 and other amphibian LEAP2 mature peptides

**Fig. 3:**
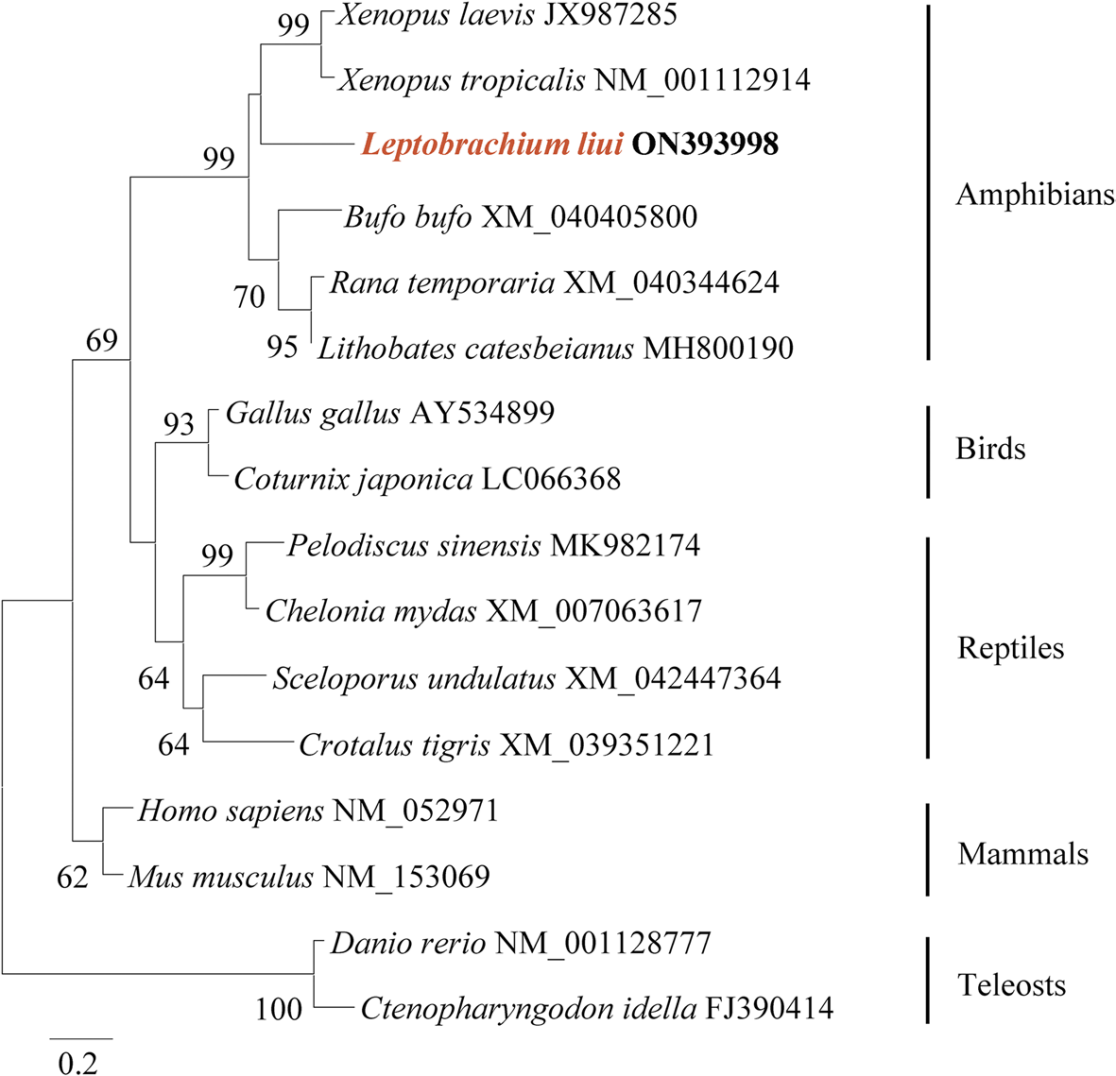
Phylogenetic reconstruction of amino acid sequences of LEAP2 based on neighbour-joining method. The values at the forks indicate the percentage of trees in which this grouping occurred after bootstrapping (1000 replicates; shown only when > 60%). The scale bar shows the number of substitutions per base

The cDNA sequence of *Ll-LEAP2* was deposited to the GenBank database (https://www.ncbi.nlm.nih.gov) under accession No. ON393998. It was estimated that the open reading frame of *Ll-LEAP2* consists of 80 amino acids and is 243 nt in length. The Ll-LEAP2 contained three parts: a signal peptide, a prodomain, and a mature peptide (Fig. 1). The third part of Ll-LEAP2 was 4.5 kDa in putative molecular weight and 8.79 in *p*I. The result of Ll-LEAP2 alignment with other amphibian LEAP2 signifies the highly conserved nature of the mature peptide, but the signal peptide and prodomain were subjected to greater variability. The mature Ll-LEAP2 peptide contains five cysteine residues, four of which are conserved among amphibian LEAP2 homologs. Further analyses of the amphibian LEAP2 mature peptide revealed a mostly helical secondary structure (Fig. 2). Based on the phylogenetic tree, amphibian LEAP2s were discovered to be clustered and distinguished from other vertebrate species, and Ll-LEAP2 was closest to the LEAP2 found in the Tropical Clawed Frog (*Xenopus tropicalis*) (Fig. 3). The highly conserved nature of the mature peptide of Ll-LEAP2 strongly suggests its importance in vertebrates despite different evolutionary history.

### Protein concentration and gene expression

**Fig. 4:**
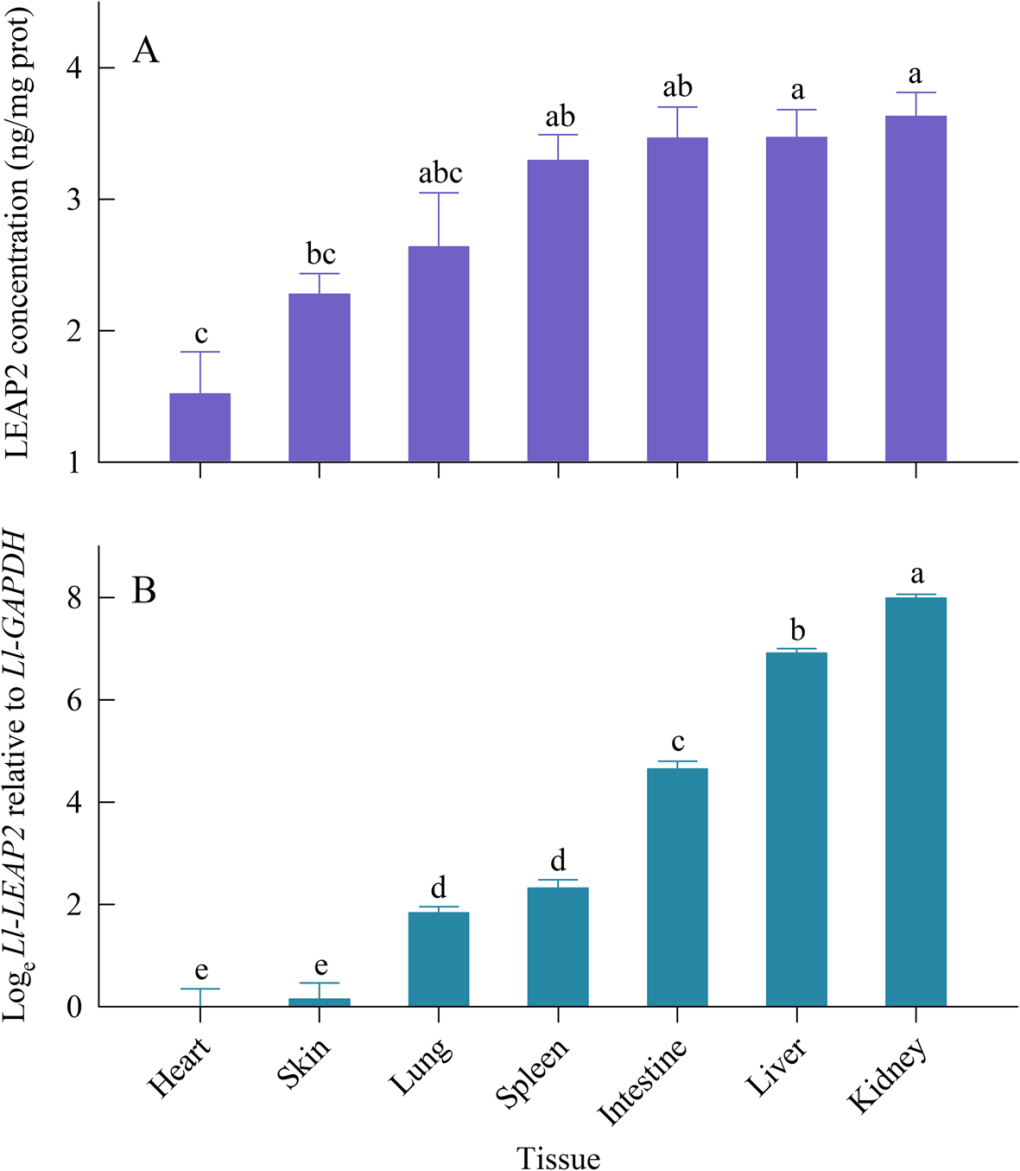
Mean values (+ SE) for (A) LEAP concentration and (B) its gene relative expression of different tissues in healthy *Leptobrachium liui*. Means with different letters differ significantly (Tukey’s post hoc test, a > b > c > d > e)

The LEAP2 concentrations differed significantly in the seven tissues (ANOVA, *F*_6, 21_ = 9.18, *P* < 0.001). The mean LEAP2 concentration was highest in the kidney and liver and lowest in the heart, with the other tissues in between (Fig. 4A). *Ll-LEAP2* was found to be expressed in all tissues (ANOVA, *F*_6, 21_ = 234.69, *P* < 0.001), with the highest expression in kidney, followed by liver, intestine, and spleen, and the lowest in heart and skin (Fig. 4B). The level of gene expression in the kidney was 2487.9-fold compared to the heart. Our results indicated that the *Ll-LEAP2* is ubiquitously expressed in *L. liui* and highly expressed in the kidney. Similarly, the protein concentration of LEAP2 supported the mRNA expression level result. Hence, there was a corresponding increase in LEAP2 concentration when there was higher expression of *Ll-LEAP2.*

### Changes in Ll-LEAP2 expression post *Aeromonas hydrophila* infection

**Fig. 5:**
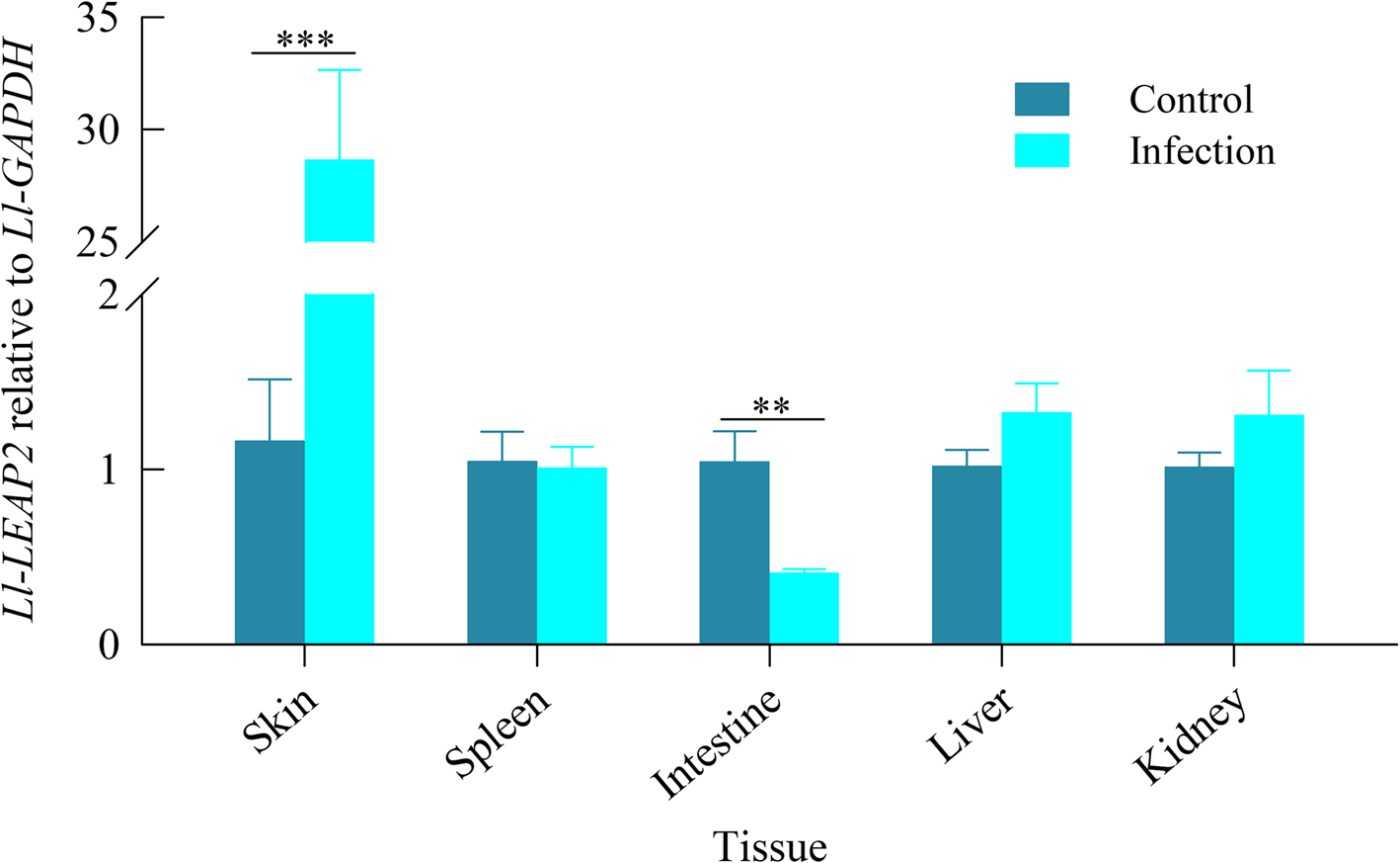
Mean values (+ SE) for the relative expression of *Ll-LEAP2* between control and infection groups in different tissues. **: *P* < 0.01, ***: *P* < 0.001

We found significant differences in *Ll-LEAP2* expression levels between the control and infection groups in the skin and intestine (*t*-test, both *P* < 0.01), with a 24.7-fold up-regulation in the skin of the infection group, and a 2.6-fold down-regulation in the intestine of the infection group (Fig. 5). However, there was no significant difference in expression levels between control and infection group for the rest of the other tissues organs which includes the liver, kidney, and spleen (*t*-test, all *P* > 0.05). In conclusion, the expression of *Ll-LEAP2* was significantly altered only in the skin and intestine after *A. hydrophila* infection.

### In vitro antibacterial activity

As shown in Table 2, antimicrobial activities of the functionally mature peptide Ll-LEAP2 and other studied Sm-LEAP2 from rockfish (*Sebastiscus marmoratus*) [23] were listed. The strongest antimicrobial activity of Ll-LEAP2 was towards *E. coli* and *S. enterica*, with an MIC value of 2.78 μM. The MIC value of Ll-LEAP2 against *A. hydrophila* and *S. aureus* was 5.6 μM, and those against *P. aeruginosa* was 22.2 μM. However, we observed no significant bactericidal activity against *P. mirabilis*, *S. sonnei*, *V. alginolyticus*, and *V. parahaemolyticus* at the set concentrations of Ll-LEAP2. In summary, Ll-LEAP2 had the effective antibacterial activity against gram-positive and negative bacteria, and the MIC values of Ll-LEAP2 were all lower than those of Sm-LEAP2 in the same tested bacteria (Table 2).

### Effects on the cell membrane integrity

**Fig. 6:**
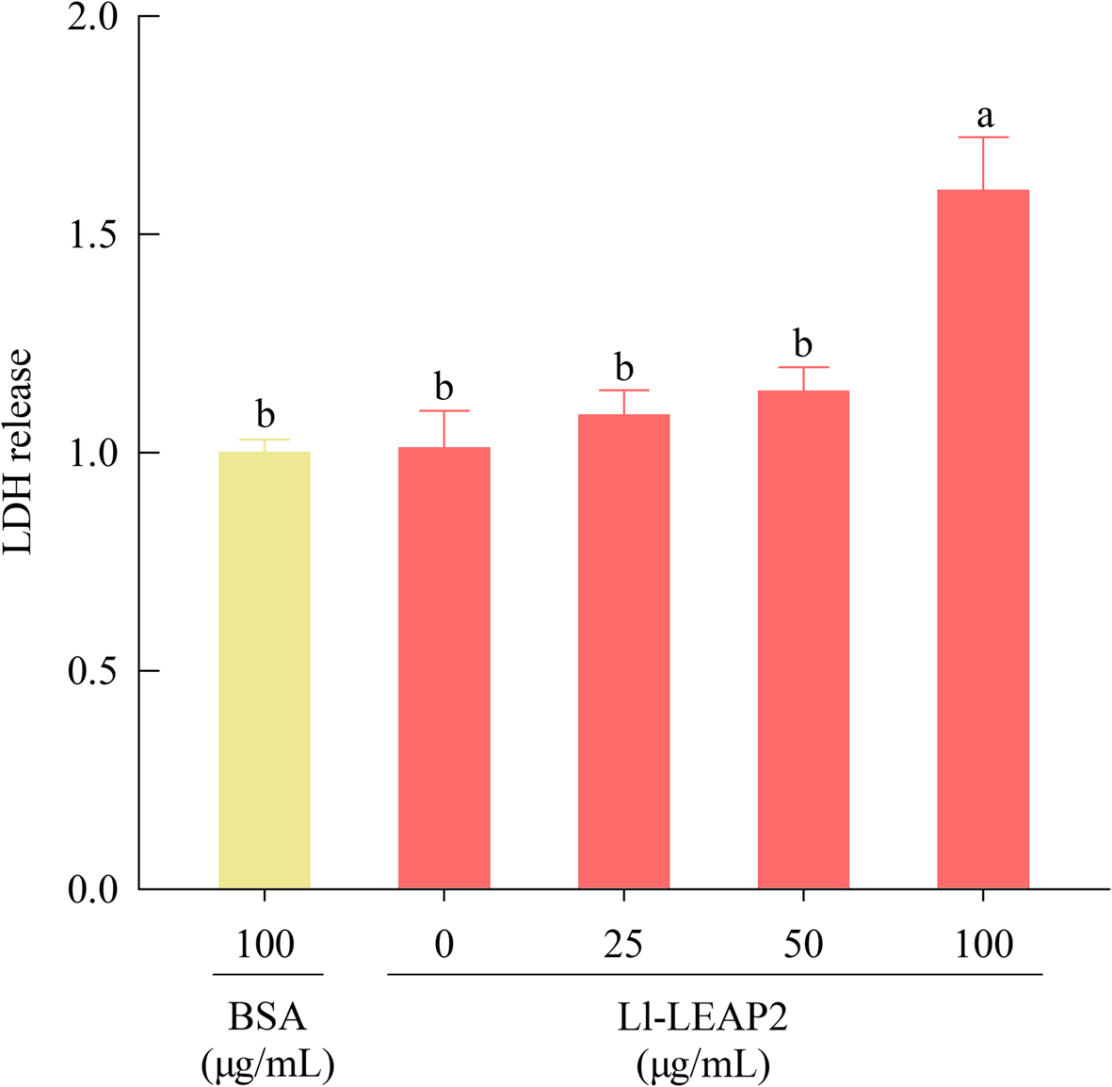
Effects of Ll-LEAP2 on the integrity of cell membrane in *Aeromonas hydrophila*. The BSA treatment was used as the negative control group. LDH release represents fold-change relative to the control group, which was assigned a value of 1. Means with different letters indicate significant differences (Tukey’s *post hoc* test, a > b)

Ll-LEAP2 significantly affected the integrity of cell membrane in *A. hydrophila* (ANOVA, *F*_4, 15_ = 10.44, *P* < 0.001). A significant amount of LDH was detected as the concentration of Ll-LEAP2 reached 100 μg/mL, which was 1.64-fold that of the BSA treatment (Tukey’s post hoc test, *P* < 0.001) (Fig. 6), indicating that LI-LEAP2 induces disruption of cell membrane in *A. hydrophila.* The membrane disruption concentration of Ll-LEAP2 was higher than the MIC, suggesting that Ll-LEAP2 had multiple antibacterial mechanisms against *A. hydrophila*, possibly including membrane disruption and gDNA hydrolysis.

### Hydrolytic effect on bacterial gDNA

**Fig. 7:**
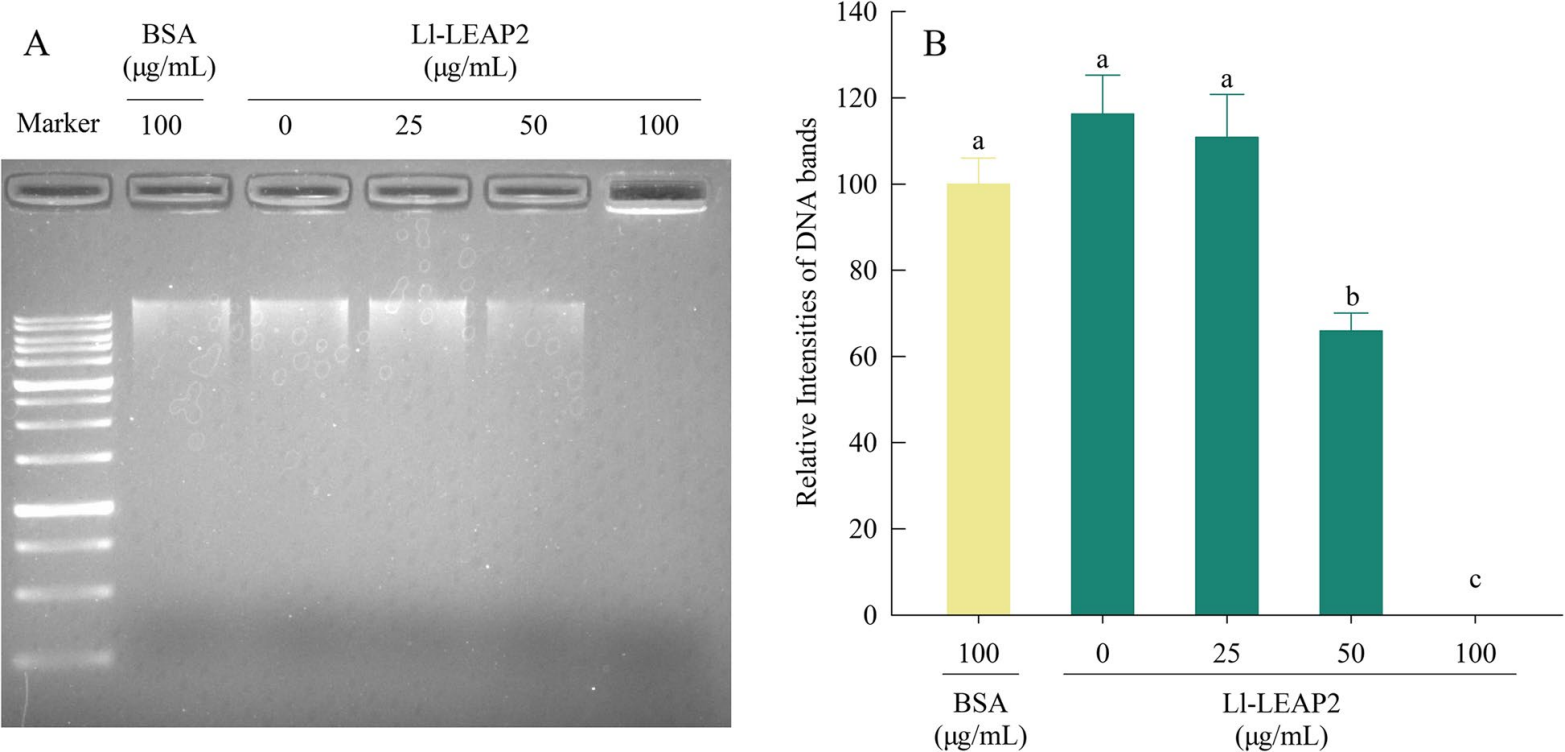
Hydrolytic effect of Ll-LEAP2 on bacterial genomic DNA. A Various concentrations of Ll-LEAP2 were incubated with 800 ng of genomic DNA of *Aeromonas hydrophila* at room temperature for 30 min, then genomic DNA was analyzed by electrophoresis on a 1.0% agarose gel. The BSA treatment was used as the negative control group. One of three independent experiments is shown. B Mean values (+SE) for the intensity of nucleic acid bands in different treatments. Means with different letters indicate significant differences (Tukey’s *post hoc* test, a > b > c)

Ll-LEAP2 significantly hydrolyzed bacterial gDNA (ANOVA, *F*_4, 15_ = 49.48, *P* < 0.001). When Ll-LEAP2 concentration increased, there was a corresponding decrease in the intensity of bacterial gDNA band (Fig. 7A). 36.71% of bacterial gDNA was hydrolyzed in the Ll-LEAP2 concentration of 50 μg/mL, while the bacterial gDNA was completely hydrolyzed under the 100 μg/mL Ll-LEAP2 treatment (Tukey’s post hoc test, both *P* < 0.001) (Fig. 7B). Below 100 μg/mL concentration, the effect of Ll-LEAP2 was stronger in gDNA hydrolysis than in membrane disruption, and the synergistic effect of the two or more bacteriostatic effects on *A. hydrophila* resulted in a low MIC value of Ll-LEAP2.

## Discussion

LEAP2 is a small cysteine-rich cationic AMP that is necessary for functioning of the immune system [15]. In this study, we identified the cDNA sequence of a putative *Ll-LEAP2*, and predicted that Ll-LEAP2 consists of three parts: a signal peptide, a prodomain, and a mature peptide. To date, there are currently no studies on the function of the prodomain of LEAP2, or the propeptide of LEAP2. However, cathelicidin, a type of AMPs, which has the similar prodomain structure as LEAP2, showed no antimicrobial function in its prodomain [24]. However, the cathelicidin propeptide has stronger antimicrobial activity than the mature peptide when targeting certain gram-negative bacteria [24]. Other studies have also shown that the propeptide of AMPs have certain functions like the *Drosophila* Baramicin A, which is an antifungal peptide [8], hence more work is needed to further determine the various functions that LI-LEAP2 may possess.

Lastly, the mature peptide of Ll-LEAP2 has four conserved cysteines, as reported in the African clawed frog [22]. The analysis of the secondary structure of the mature peptide of Ll-LEAP2 revealed that most of its secondary structure was helical. In the human LEAP2 structure, the mature peptide is known to have a compact central core with disorder at the N and C termini [15]. The core comprises of a β-hairpin and a 3_10_-helix which are braced by two intramolecular-disulfide bonds and further stabilized by a network of hydrogen bonds [15]. According to the phylogenetic analysis, Ll-LEAP2 belongs to the amphibian LEAP2 group and is closest to the LEAP2 in *X. tropicalis*.

*Ll-LEAP2* was constitutively expressed in many healthy tissues mainly in the kidney and liver, and these results are congruent with other vertebrates studies which showed that *LEAP2* was highly expressed in these tissues [15, 18, 25, 26]. Meanwhile, our results confirmed that tissues with higher *Ll-LEAP2* expression also had a higher concentration of the corresponding peptide in *L. liui*. Additionally, our results showed that *Ll-LEAP2* expression was significantly up-regulated in skin tissues following *A. hydrophila* infection and this was similar to other studies [27, 28]. In Yang et al. [28], the expression of *LEAP2* in the common carp was swiftly up-regulated in the skin after infections with *Vibrio anguillarum*. Meanwhile, the expression of *Ll-LEAP2* was down-regulated in the intestine, which was similar to other results reported in the mudskipper [20] and large yellow croaker [29]. As the intestinal immune system is reliant on a rich and healthy microbiota, AMPs are found to play a key role in establishing and maintaining a stable gut microbiota for the organism [30]. After bacterial infection, the AMPs concentrations may be altered with some being up-regulated while others are being down-regulated. This results in a new equilibrium of AMPs concentration in response to infection by pathogenic microorganisms and creates a more robust gut microbiota [3].

Recently, several in vitro studies have shown that vertebrate LEAP2s have direct antibacterial activity [15, 17, 20, 31, 32]. Synthetic peptides of the mudskipper LEAP2 demonstrated antimicrobial activity against *V. alginolyticus* and *V. vulnificus*, in vitro [20]. Despite this, there is no information available on the antibacterial properties of amphibian LEAP2s. We observed that Ll-LEAP2 had significant antimicrobial activity against five tested bacteria (*A. hydrophila*, *E. coli*, *P. aeruginosa*, *S. enterica*, and *S. aureus*). Moreover, the MIC of 2.78 μM for Ll-LEAP2 against *S. enterica* and *E. coli* was considered to be relatively low, suggesting that this peptide can potentially inhibit *S. enterica* and *E. coli* infections. Although our study showed no antimicrobial activity of Ll-LEAP2 against *Pichia pastoris*; in another study, LEAP2 was effective in inhibiting yeast growth [23]. Specifically, *S. marmoratus* LEAP2 displayed antibacterial activity against *Candida albicans* and *Cryptococcus neoformans* [23]. Furthermore, we investigated the bactericidal mechanism of Ll-LEAP2 and found that Ll-LEAP2 can disrupt bacterial cell membranes and hydrolyze bacterial gDNA, which is consistent with the results observed in mammals [17, 33] and fish [25].

## Conclusion

In summary, a LEAP2 homologue from *L. liui* was characterized, and its protein concentration and gene expression were higher in the kidney and liver of *L. liui*. Ll-LEAP2 was revealed to possess antibacterial activity against a wide range of bacteria species including both gram-positive and negative bacteria. Meanwhile, Ll-LEAP2 has the ability to kill specific bacteria by disrupting bacterial membrane and hydrolyzing bacterial gDNA. With more to uncover, the immunomodulatory functions and wound-healing activities of Ll-LEAP2 holds great potential for future research.

## Materials and methods

### Animal collection and management

All the samples were collected with permission in accordance with the local license. All methods were performed in accordance with the relevant guidelines and regulations in the ethics approval and consent to participate section along with the approval of the Ethics Committee of Lishui University (Permit No. AREC-LSU202011–001) and ARRIVE guidelines.

Twelve male adult *L. iui* individuals (30–40 g/individual) were captured from the Zhejiang Jiulongshan National Nature Reserve (N28.370°, E118.887°) in Suichang, China on 12 November 2020, and randomly assigned to control and experimental groups in this study. During the breeding season, they are defined by having cornified spies in the maxillary of males and sexual size dimorphism in which the males are generally larger than the females. Each toad was placed separately in a covered food-grade polypropylene plastic bin (300 × 200 × 120 mm) with 2.4 L pure water free from pathogens at 9–12 °C in a system with recirculating filtered water and had 2 weeks to be accustomed to laboratory conditions before the actual start of the experiments.

### Molecular characterization of Ll-LEAP2 cDNA

*Ll-LEAP2* cDNA was assembled using the transcriptome of *L. liui* liver deposited in the NCBI SRA database (https://www.ncbi.nlm.nih.gov/sra) under accession No. SRR23238843, and reverse transcription-polymerase chain reaction (RT-PCR) was used to amplify the gene. The primers used were F: 5′-AGCCTGAGCTTTCAGGACTAGC-3′, R: 5′-CCTCCTTTTCCAAGCACCAG-3′, which were designed by Primer Primer 5 [34], and sequenced using Sanger Sequencing to verify the base composition of *Ll-LEAP2*. We used SignalP 5.0 [35] to predict the cleavage sites of the signal peptide, ran ClustalW [36] to analyze multiple alignments, and used PSIPRED software to predict the secondary structure of the protein, and operated MEGA version X [37] to perform phylogenetic analyses.

### Ll-LEAP2 expression profiles

After 2 weeks of animal acclimatization, four healthy individuals were dissected immediately on the ice after euthanasia with MS-222 (400 ppm), and seven tissues (spleen, lung, kidney, intestine, skin, heart, and liver) were isolated separately, transferred into a 1.5 mL plastic tube, and stored at − 80 °C. To detect the impact of pathogenic microbes on the expression of *Ll-LEAP2*, we performed *Aeromonas hydrophila* infection based on previous studies [38]. *A. hydrophila* was re-suspended in sterile 0.65% saline with a final concentration of 1 × 10^5^ colony forming units (CFU)/mL, and a single intraperitoneal injection (100 μL) was performed to all four healthy individuals in the infection group. The other four individuals were injected with the same volume of 0.65% saline as the control group. Five tissues (spleen, kidney, intestine, skin, and liver) of each individual were isolated separately and stored at − 80 °C at 12 hours post-infection until analysis. Animal dissection were done on the clean bench in experimental units of Laboratory of Amphibian Diversity Investigation at Lishui University, China.

### Biochemical assay

The concentrations of LEAP2 in seven tissues of healthy individuals were determined by enzyme-linked immunosorbent assay (ELISA) via Frog LEAP2 ELISA Kit (no.SU-B85013; Quanzhou Kenuodi Biotechnology Co., Ltd., Quanzhou, China) following the instruction of the manufacturer. Before the ELISA, tissue homogenates were prepared in advance. Each tissue was mixed with ice-cold 0.65% saline at a weight: volume ratio of 0.1 g: 9 mL, homogenized on ice, and then centrifuged at 2000 rpm at 4 °C for 10 min to precipitate insoluble tissue fragments. The supernatant was transferred into a 1.5 mL plastic tube, and centrifuged at 13,000 rpm at 4 °C for 20 min to remove additional organelles (mitochondria). The supernatant after the second centrifugation was further transferred to a 1.5 mL tube for biochemical assay. The totoal protein concentration in each sample was measured based on the Bradford method using the Total Protein Quantitative Assay Kit (no. A045–2-2; Nanjing Jiancheng Bioengineering Institute, Nanjing, China) according to the manufacturer’s instruction. The LEAP2 concentration was expressed as mass per microgram of total protein.

### Real-time quantitative PCR (qPCR)

Total RNA of each sample was extracted using Trizol reagent (Sangon Biotech, Shanghai, China) and synthesized cDNA using PrimeScript RT reagent Kit with gDNA Eraser (TaKaRa, Dalian, China). For the baseline reference gene, we used *L. liui* glyceraldehyde-3-phosphate dehydrogenase (*Ll-GAPDH*). qPCR was performed with the TB Green Premix Ex Taq (TaKaRa, Dalian, China), and data was generated using the Real-time PCR Detection System (CFX96, Bio-Rad, Hercules, CA, USA) to determine the cycle threshold (Ct) values of *Ll-LEAP2* and *Ll-GAPDH*. We used the following conditions for amplification: 95 °C for 5 min, followed by 40 cycles of 95 °C for 30 s, 60 °C for 30 s, and 72 °C for 30 s. The primers (Table 1) were designed by employing Primer3web v 4.1.0 (https://primer3.ut.ee; accessed on 01 May 2021). The 2^-ΔΔCt^ method [39] was used to calculate the relative expression of *Ll-LEAP2* to *Ll-GAPDH*. Melt curve analysis was used to determine the quality and specificity of the amplified products.

**Table 1:** Sequences of oligonucleotide primers used in this study

Gene	Accession number	Primers	Sequence (5′-3′)	Amplicon size (bp)
*Ll-LEAP2*	ON393998	LEAP-2-t(+)	ATCTTCAGCCTGGGAGATGG	154
		LEAP-2-t(−)	ATAGGACGCAGAGAAAGCCC	
*Ll-GAPDH*	ON462259	GAPDH-t(+)	AGCCGCACAGAACATCATTC	241
		GAPDH-t(−)	AACCTCGTCCTCAGTGTAGC	

### Antibacterial assay

The fully mature peptide of Ll-LEAP2 (MTPFWRGLSLRPIGASCRDASECLTQLCKKNRCCLQTFAD) was synthesized chemically (> 95% purity; Sangon Biotech, Shanghai, China). To test the antibacterial activity of Ll-LEAP2, we used a range of bacteria including the commensal bacteria *Escherichia coli*, *Salmonella enterica*, and *Shigella sonnei*, the human opportunistic pathogens *A. hydrophila*, *Listeria monocytogenes*, *Proteus mirabilis*, *Pseudomonas aeruginosa*, *Staphylococcus aureus*, *Vibrio alginolyticus*, *Vibrio parahaemolyticus*. The methylotrophic yeast *Pichia pastoris* was also part of the experiment. We used a modified two-fold microdilution method [4] to determine the minimal inhibitory concentration (MIC) of Ll-LEAP2 in eight bacterial species and one fungus. Six concentrations including 22.2 μM (100 μg/mL), 11.1 μM (50 μg/mL), 5.6 μM (25 μg/mL), 2.78 μM (12.5 μg/mL), 1.39 μM (6.25 μg/mL), and 0.69 μM (3.125 μg/mL) of Ll-LEAP2 solution were obtained using phosphate-buffered saline (PBS, pH = 7.4) as a solvent. Each microbe was diluted to 1 × 10^5^ CFU/mL in suitable media (Table 2) after incubation to the mid-logarithmic phase. We mixed 10 μL Ll-LEAP2 solution and 90 μL microbial solution and added them to the corresponding well of the 96-well plate. According to each of the appropriate incubation temperatures for each microbe, these plates were placed for 12 h in the incubators at a constant temperature of either 28 °C, 30 °C or 37 °C. Then, MIC was determined at 600 nm using Microplate Absorbance Reader (Bio-Rad, Hercules, CA, USA).

**Table 2:** Minimal inhibitory concentration (MIC) of LEAP2 against bacteria and fungus

Bacteria/fungus	Strains	Medium	Temperature (°C)	MIC (μM)	
				Ll-LEAP2	Sm-LEAP2 [23]
*Aeromonas hydrophila*	ATCC7966	LB	37	5.6	> 50
*Escherichia coli*	K12	LB	37	2.78	25–50
*Listeria monocytogenes*	ATCC19115	BHI	37	–	NT
*Pichia pastoris* (fungus)	GS115	YPD	30	–	NT
*Proteus mirabilis*	ATCC25933	LB	37	–	NT
*Pseudomonas aeruginosa*	ATCC27853	LB	37	22.2	25–50
*Salmonella enterica*	ATCC13076	LB	37	2.78	NT
*Shigella sonnei*	ATCC25931	LB	37	–	NT
*Staphylococcus aureus*	ATCC6538	LB	37	11.1	> 50
*Vibrio alginolyticus*	ATCC17749	TSB	28	–	> 50
*Vibrio parahaemolyticus*	ATCC33847	TSB	28	–	> 50

–: no inhibition detected at 22.2 μM. *NT* not detected

### Lactate dehydrogenase (LDH) release assay

Detection of damage in the bacterial cell membrane was determined by the LDH Release Assay Kit (Beyotime, Shanghai, China) following the study of Chen et al. [40]. 1 × 10^9^ CFUs of *A. hydrophila* was obtained by centrifugation at 5000×*g* for 2 min, and re-suspended into 200 μL PBS. Subsequently 200 μL of 5 × 10^9^ CFU/mL bacterial solution was mixed with Ll-LEAP2 to create a final concentration of 0, 25, 50, and 100 μg/mL, respectively, and followed by incubation at 37 °C for 2 h. In the negative control group, bovine serum albumin (BSA)/PBS solution with a final concentration of 100 μg/mL was added to the 200 μL bacterial solution. We separated the supernatant by centrifugation at 8000×*g* for 2 min and added 120 μL to each well of the 96-well plate. A total of 60 μL of LDH detection solution was then added to each well and incubated at room temperature (approximately 23–25 °C) for 30 min, and its absorbance was measured at 490 nm using Microplate Absorbance Reader (Bio-Rad, Hercules, CA, USA).

### DNA degradation assay

The hydrolytic effect of Ll-LEAP2 on bacterial genomic DNA (gDNA) was measured based on the previous method of Chen et al. [41]. We used the Ezup Column Bacteria Genomic DNA Purification Kit (Sangon Biotech, Shanghai, China) to extract *A. hydrophila* gDNA. The concentration of gDNA was determined using a microvolume spectrophotometer (NanoDrop 2000, Thermo Fisher Scientific, Wilmington, DE, USA). Subsequently, a total of 800 ng of gDNA was added to 20 μL of PBS with 0, 25, 50, and 100 μg/mL of Ll-LEAP2, respectively. In the negative control group, 20 μL BSA/PBS solution (100 μg/mL) was mixed with 800 ng of gDNA. After incubating at room temperature (approximately 23–25 °C) for 30 min, 4 μL of 6 × loading buffer was mixed with the sample. Each reaction was analyzed by 1.0% agarose gel electrophoresis in 0.5 × tris-acetate-EDTA buffer and electrophoretic scanning. The intensity of each nucleic acid band was determined using ImageJ (NIH, Bethesda, MD, USA).

### Statistical analysis

All results were presented as mean ± standard error (SE), and differences were considered to be statistically significant at *P* < 0.05. Prior to analysis, the normality and homogeneity of the data were verified using Kolmogorov-Smirnov and Bartlett’s tests, respectively. The data of tissue-specific expression and relative expression between control and infection groups in skin and intestine tissues were log_e_-transformed and fulfilled the normality assumptions for parametric tests. One-way analyses of variance (ANOVA) was used to examine the differences in LEAP2 protein concentration and its gene expression among different tissues and the effect of Ll-LEAP2 on the integrity of membrane and gDNA hydrolysis in *A. hydrophila*. Tukey’s *post hoc* test was performed on the traits that differed among the different tissues and treatments. We used the *t*-test to examine whether the relative expression of *Ll-LEAP2* in each tissue was different between the control and infection groups. The statistical analyses were performed using SPSS Version 13.0 statistical software (SPSS Inc., Chicago, IL, USA).

## Data Availability

The sequencing data supporting this study are openly available in NCBI SRA database (https://www.ncbi.nlm.nih.gov/sra/SRR23238843). The datasets generated and/or analyzed during the current study are available in the ResearchGate (www.researchgate.net) (10.13140/RG.2.2.18035.40483). Further inquiries can be directed to the corresponding author.

## Abbreviations

AMP: Antimicrobial peptide
ANOVA: Analyses of variance
BSA: bovine serum albumin
CFU: Colony forming units
Ct: Cycle threshold
ELISA: Enzyme-linked immunosorbent assay
GAPDH: Glyceraldehyde-3-phosphate dehydrogenase
gDNA: Genomic DNA
LDH: Lactate dehydrogenase
LEAP2: Liver-expressed antimicrobial peptide 2
MIC: Minimal inhibitory concentration
PBS: Phosphate-buffered saline
qPCR: Real-time quantitative PCR
RT-PCR: Reverse transcription-polymerase chain reaction

## References

[1] Patocka J, Nepovimova E, Klimova B, Wu Q, Kuca K (2019). Antimicrobial peptides: amphibian host defense peptides. Curr Med Chem.

[2] Hancock RE, Nijnik A, Philpott DJ (2012). Modulating immunity as a therapy for bacterial infections. Nat Rev Microbiol.

[3] Zhu QY, Chen RY, Yu J, Ding GH, Seah RWX, Chen J (2022). Antimicrobial peptide hepcidin contributes to restoration of the intestinal flora after *Aeromonas hydrophila* infection in *Acrossocheilus fasciatus*. Comp Biochem Physiol C Toxicol Pharmacol.

[4] Yu SS, Zhao ZH, Gong XF, Fan XL, Lin ZH, Chen J (2022). Antimicrobial and immunomodulatory activity of beta-defensin from the Chinese spiny frog (*Quasipaa spinosa*). Dev Comp Immunol.

[5] Fan XL, Yu SS, Zhao JL, Li Y, Zhan DJ, Xu F, Lin ZH, Chen J (2022). Brevinin-2PN, an antimicrobial peptide identified from dark-spotted frog (*Pelophylax nigromaculatus*), exhibits wound-healing activity. Dev Comp Immunol.

[6] Torrent M, Pulido D, Rivas L, Andreu D (2012). Antimicrobial peptide action on parasites. Curr Drug Targets.

[7] Myers AN, Lawhon SD, Diesel AB, Bradley CW, Rodrigues Hoffmann A, Murphy WJ (2022). An ancient haplotype containing antimicrobial peptide gene variants is associated with severe fungal skin disease in *Persian* cats. PLoS Genet.

[8] Hanson MA, Cohen LB, Marra A, Iatsenko I, Wasserman SA, Lemaitre B. The *Drosophila Baramicin* polypeptide gene protects against fungal infection. Cold Spring Harbor Laboratory. 2021. 10.1101/2020.11.23.394148. 10.1371/journal.ppat.1009846 PMC8423362 34432851

[9] Rollins-Smith LA, Conlon JM (2005). Antimicrobial peptide defenses against chytridiomycosis, an emerging infectious disease of amphibian populations. Dev Comp Immunol.

[10] Field KA, Johnson JS, Lilley TM, Reeder SM, Rogers EJ, Behr MJ, Reeder DM (2015). The white-nose syndrome transcriptome: activation of anti-fungal host responses in wing tissue of hibernating little brown myotis. PLoS Pathog.

[11] Brown KL, Hancock RE (2006). Cationic host defense (antimicrobial) peptides. Curr Opin Immunol.

[12] Mookherjee N, Anderson MA, Haagsman HP, Davidson DJ (2020). Antimicrobial host defence peptides: functions and clinical potential. Nat Rev Drug Discov.

[13] Demori I, Rashed ZE, Corradino V, Catalano A, Rovegno L, Queirolo L, Salvidio S, Biggi E, Zanotti-Russo M, Canesi L, Catenazzi A, Grasselli E (2019). Peptides for skin protection and healing in amphibians. Molecules.

[14] Di Grazia A, Cappiello F, Imanishi A, Mastrofrancesco A, Picardo M, Paus R, Mangoni ML (2015). The frog skin-derived antimicrobial peptide esculentin-1a(1-21)NH2 promotes the migration of human HaCaT keratinocytes in an EGF receptor-dependent manner: a novel promoter of human skin wound healing?. PLoS One.

[15] Krause A, Sillard R, Kleemeier B, Kluver E, Maronde E, Conejo-García JR, Forssmann WG, Schulz-Knappe P, Nehls MC, Wattler F, Wattler S, Adermann K (2003). Isolation and biochemical characterization of LEAP-2, a novel blood peptide expressed in the liver. Protein Sci.

[16] Henriques ST, Tan CC, Craik DJ, Clark RJ (2010). Structural and functional analysis of human liver-expressed antimicrobial peptide 2. ChemBioChem.

[17] Hocquellet A, Odaert B, Cabanne C, Noubhani A, Dieryck W, Joucla G, Le Senechal C, Milenkov M, Chaignepain S, Schmitter JM, Claverol S, Santarelli X, Dufourc EJ, Bonneu M, Garbay B, Costaglioli P (2010). Structure-activity relationship of human liver-expressed antimicrobial peptide 2. Peptides.

[18] Ishige T, Hara H, Hirano T, Kono T, Hanzawa K (2016). Characterization and expression of non-polymorphic liver expressed antimicrobial peptide 2: LEAP-2 in the Japanese quail, *Coturnix japonica*. Anim Sci J.

[19] van Hoek ML. Antimicrobial peptides in reptiles. Pharmaceuticals (Basel). 2014;7:723–753. 10.3390/ph7060723. 10.3390/ph7060723 PMC4078517 24918867

[20] Chen J, Chen Q, Lu XJ, Chen J (2016). The protection effect of LEAP-2 on the mudskipper (*Boleophthalmus pectinirostris*) against *Edwardsiella tarda* infection is associated with its immunomodulatory activity on monocytes/macrophages. Fish Shellfish Immunol.

[21] Liu B, Liu GD, Guo HY, Zhu KC, Guo L, Zhang N, Liu BS, Jiang SG, Zhang DC (2020). Characterization and functional analysis of liver-expressed antimicrobial peptide-2 (LEAP-2) from golden pompano *Trachinotus ovatus* (Linnaeus 1758). Fish Shellfish Immunol.

[22] Thiébaud P, Garbay B, Auguste P, Sénéchal CL, Maciejewska Z, Fédou S, Gauthereau X, Costaglioli P, Thézé N (2016). Overexpression of Leap2 impairs *Xenopus* embryonic development and modulates FGF and activin signals. Peptides.

[23] Bo J, Yang Y, Zheng RH, Fang C, Jiang YL, Liu J, Chen MY, Hong FK, Bailey C, Segner H, Wang KJ. Antimicrobial activity and mechanisms of multiple antimicrobial peptides isolated from rockfish Sebastiscus marmoratus. Fish Shellfish Immunol. 2019;93:1007–17. 10.1016/j.fsi.2019.08.054. 10.1016/j.fsi.2019.08.054 31449978

[24] Pazgier M, Ericksen B, Ling M, Toth E, Shi J, Li X, Galliher-Beckley A, Lan L, Zou G, Zhan C, Yuan W, Pozharski E, Lu W (2013). Structural and functional analysis of the pro-domain of human cathelicidin, LL-37. Biochemistry.

[25] Li HX, Lu XJ, Li CH, Chen J (2015). Molecular characterization of the liver-expressed antimicrobial peptide 2 (LEAP-2) in a teleost fish, *Plecoglossus altivelis*: antimicrobial activity and molecular mechanism. Mol Immunol.

[26] Liu T, Gao Y, Wang R, Xu T (2014). Characterization, evolution and functional analysis of the liver-expressed antimicrobial peptide 2 (LEAP-2) gene in miiuy croaker. Fish Shellfish Immunol.

[27] Liu F, Li JL, Yue GH, Fu JJ, Zhou ZF (2010). Molecular cloning and expression analysis of the liver-expressed antimicrobial peptide 2 (LEAP-2) gene in grass carp. Vet Immunol Immunopathol.

[28] Yang G, Guo H, Li H, Shan S, Zhang X, Rombout JH, An L (2014). Molecular characterization of LEAP-2 cDNA in common carp (*Cyprinus carpio* L.) and the differential expression upon a *vibrio anguillarum* stimulus; indications for a significant immune role in skin. Fish Shellfish Immunol.

[29] Li HX, Lu XJ, Li CH, Chen J (2014). Molecular characterization and functional analysis of two distinct liver-expressed antimicrobial peptide 2 (LEAP-2) genes in large yellow croaker (*Larimichthys crocea*). Fish Shellfish Immunol.

[30] Bosch TCG, Zasloff M (2021). Antimicrobial peptides——or how our ancestors learned to control the microbiome. mBio.

[31] Kim CH, Kim EJ, Nam YK (2019). Subfunctionalization and evolution of liver-expressed antimicrobial peptide 2 (LEAP2) isoform genes in Siberian sturgeon (*Acipenser baerii*), a primitive chondrostean fish species. Fish Shellfish Immunol.

[32] Luo SW, Luo KK, Liu SJ (2020). A novel LEAP-2 in diploid hybrid fish (*Carassius auratus cuvieri* ♀ ×*Carassius auratus* red var. ♂) confers protection against bacteria-stimulated inflammatory response. Comp Biochem Physiol C: Toxicol Pharmacol.

[33] Townes CL, Michailidis G, Hall J (2009). The interaction of the antimicrobial peptide cLEAP-2 and the bacterial membrane. Biochem Biophys Res Commun.

[34] Lalitha S (2000). Primer Premier 5. Biotech Softw Internet Rep.

[35] Almagro Armenteros JJ, Tsirigos KD, Sønderby CK, Petersen TN, Winther O, Brunak S, von Heijne G, Nielsen H (2019). SignalP 5.0 improves signal peptide predictions using deep neural networks. Nat Biotech.

[36] Thompson JD, Higgins DG, Gibson TJ (1994). CLUSTAL W: improving the sensitivity of progressive multiple sequence alignment through sequence weighting, position-specific gap penalties and weight matrix choice. Nucleic Acids Res.

[37] Kumar S, Stecher G, Li M, Knyaz C, Tamura K (2018). MEGA X: molecular evolutionary genetics analysis across computing platforms. Mol Biol Evol.

[38] Jiang W, Chen J, Guo ZP, Zhang L, Chen GP (2020). Molecular characterization of a MOSPD2 homolog in the barbel steed (*Hemibarbus labeo*) and its involvement in monocyte/macrophage and neutrophil migration. Mol Immunol.

[39] Livak KJ, Schmittgen TD (2001). Analysis of relative gene expression data using real-time quantitative PCR and the 2(−Delta Delta C(T)) method. Methods.

[40] Chen J, Lin YF, Chen JH, Chen X, Lin ZH (2021). Molecular characterization of cathelicidin in tiger frog (*Hoplobatrachus rugulosus*): antimicrobial activity and immunomodulatory activity. Comp Biochem Physiol C: Toxicol Pharmacol.

[41] Chen J, Nie L, Chen J (2018). Mudskipper (*Boleophthalmus pectinirostris*) Hepcidin-1 and Hepcidin-2 present different gene expression profile and antibacterial activity and possess distinct protective effect against *Edwardsiella tarda* infection. Probiotics Antimicrob Proteins.

